# *In Vitro* Expansion of Bone Marrow Derived Mesenchymal Stem Cells Alters DNA Double Strand Break Repair of Etoposide Induced DNA Damage

**DOI:** 10.1155/2016/8270464

**Published:** 2016-01-06

**Authors:** Ian Hare, Marieta Gencheva, Rebecca Evans, James Fortney, Debbie Piktel, Jeffrey A. Vos, David Howell, Laura F. Gibson

**Affiliations:** ^1^Alexander B. Osborn Hematopoietic Malignancy and Transplantation Program of the West Virginia University Cancer Institute, Robert C. Byrd Health Sciences Center, West Virginia University School of Medicine, Morgantown, WV 26506, USA; ^2^Department of Microbiology, Immunology and Cell Biology, Robert C. Byrd Health Sciences Center, West Virginia University School of Medicine, Morgantown, WV 26506, USA; ^3^Department of Pathology, School of Medicine, West Virginia University, Morgantown, WV 26506, USA

## Abstract

Mesenchymal stem cells (MSCs) are of interest for use in diverse cellular therapies.* Ex vivo* expansion of MSCs intended for transplantation must result in generation of cells that maintain fidelity of critical functions. Previous investigations have identified genetic and phenotypic alterations of MSCs with* in vitro* passage, but little is known regarding how culturing influences the ability of MSCs to repair double strand DNA breaks (DSBs), the most severe of DNA lesions. To investigate the response to DSB stress with passage* in vitro*, primary human MSCs were exposed to etoposide (VP16) at various passages with subsequent evaluation of cellular damage responses and DNA repair. Passage number did not affect susceptibility to VP16 or the incidence and repair kinetics of DSBs. Nonhomologous end joining (NHEJ) transcripts showed little alteration with VP16 exposure or passage; however, homologous recombination (HR) transcripts were reduced following VP16 exposure with this decrease amplified as MSCs were passaged* in vitro*. Functional evaluations of NHEJ and HR showed that MSCs were unable to activate NHEJ repair following VP16 stress in cells after successive passage. These results indicate that* ex vivo* expansion of MSCs alters their ability to perform DSB repair, a necessary function for cells intended for transplantation.

## 1. Introduction

MSCs are a mesoderm derived stromal population defined functionally by their ability to differentiate into various cell types* in vitro*. Osteoblasts, adipocytes, and chondrocytes have been shown to arise from MSC precursors under various culture conditions [1]. MSCs have also been shown to have immunomodulatory properties, displaying the ability to suppress adaptive and innate immune responses through the secretion of anti-inflammatory cytokines [2]. In addition to these functional characteristics, MSCs are able to persist in culture, making it possible to alter gene expression of the cells through various transfection techniques. Such an approach has been utilized in a rat model of myocardial infarction to deliver HIF-1*α* expressing MSCs to damaged heart tissue [3]. These cellular attributes have made MSCs an attractive candidate for the development of stem cell therapies in humans. MSCs can be acquired from various tissues of the body including the bone marrow [4]. The injection of bone marrow derived MSCs is currently undergoing Phase III clinical trials in the United States for the treatment of Crohn's disease and myocardial infarction (clinicaltrial.gov identifiers NCT00482092 and NCT01394432, resp.). In addition, several preclinical applications have been described in animal models of disease, such as autoimmune encephalitis, graft versus host disease, rheumatoid arthritis, type I diabetes, and inflammatory bowel disease [5].

The use of MSCs for cellular therapies requires the ability of* ex vivo* expansion generating adequate numbers of cells for treatment. Although there is significant evidence documenting the clinical utility of MSCs, they are a heterogeneous population of cells that differ based on the means by which they are acquired and how they are cultured* in vitro* [6]. Given their utility, it is important to understand how they are altered during the necessary* ex vivo* expansion prior to patient administration. It has been shown previously that* ex vivo* expansion of MSCs results in alterations in genome stability [7, 8], epigenetics [9, 10], and functional abilities to differentiate into osteogenic cells [11, 12]. However, these studies have been performed in nonstressed conditions, and little has been shown regarding how the activity of MSCs may be altered once administered to a patient where they may encounter cellular stresses, such as DNA damage. Eukaryotes have evolved means to recover from many types of DNA damage, the most lethal of which are double strand breaks (DSBs). DSBs are repaired by two major repair pathways: nonhomologous end joining (NHEJ) and homologous recombination (HR) [13]. NHEJ involves the resection of nucleotides from both sides of a DSB, followed by the binding of Ku70 and Ku80 proteins with DNA-PK_cs_, which recruits DNA ligase IV and initiates ligation of the break [14]. NHEJ is capable of repairing incompatible ends regardless of cell cycle status. When a sister chromatid is present (during S/G2/M phases of cell cycle), HR is capable of repairing a break by resecting both ends of a break, followed by insertion of the 3′ resected end into the homologous sequence, thereby using it as a template for repair. Due to the presence of a homologous sequence, HR is less error-prone than NHEJ; however, the necessity of a reference template limits the utilization of HR to S/G2/M phases of cell cycle [15].

To determine how* ex vivo* expansion alters the response of MSCs to stress, we utilized etoposide (VP16), a DNA type-II topoisomerase inhibitor that specifically induces DSBs following DNA replication [16]. DSBs are the most lethal of DNA lesions, resulting in a larger degree of somatic mutation or apoptosis than other DNA lesions [17]. To determine how* ex vivo* expansion alters the response of MSCs to DSB stress, we passaged bone marrow derived MSCs* in vitro*, evaluating their responses to cellular stress and DSB repair following VP16 exposure.

## 2. Materials and Methods

### 2.1. Human MSC Isolation and Cell Culture

Bone marrow aspirates were deidentified samples from patients treated at the West Virginia University Healthcare System. Cells were cultured in *α*-MEM supplemented with 10% fetal bovine serum (BSA), 2 mM L-glutamine, 100 U/mL penicillin, and 100 *μ*g/mL streptomycin and housed at 37°C, 6% CO_2_. MSCs were derived from donors who had no previous exposure to chemotherapy or irradiation and no history of malignancy.

### 2.2. Surface Staining of MSCs

Cells were trypsinized and washed in 1x phosphate buffered saline (PBS). Primary antibodies (1 *μ*g) specific for human CD45, CD44, CD105, and CD166 (BD Pharmingen, San Jose, California, United States) were added and incubated on ice for 20 minutes in PBS/3% BSA. Cells were washed in PBS/3% BSA and then incubated with 1 *μ*g donkey anti-mouse-AF488 (BD Pharmingen, San Jose, California, United States) for 20 minutes on ice. Cells were then washed in PBS/3% BSA, resuspended in 400 *μ*L PBS, and immediately analyzed using a FACS Fortessa (BD Biosciences, Franklin Lakes, New Jersey, United States).

### 2.3. Differentiation of MSCs

To induce osteogenic differentiation in human MSC cultures, cells were plated at 90% confluence then cultured in Stempro Osteogenesis Differentiation Kit Medium (Life Technologies, Carlsbad, California, United States) for 21 days. Differentiation medium was changed every 3 days. To induce adipogenic differentiation, cells were treated similarly only cultured in Stempro Adipogenesis Differentiation Kit Medium (Life Technologies, Carlsbad, California, United States) for 10 days. Differentiated cells were compared to undifferentiated controls, cultured in normal MSC medium (see Section 2.1).

### 2.4. Alizarin Red and Oil Red Staining

Human MSCs were fixed in 4% paraformaldehyde (Sigma, St. Louis, Missouri, United States) for 4 hours prior to staining. Alizarin red and oil red staining were performed as described previously [18, 19]. Cells were washed in deionized water and imaged immediately at 100x magnification using a Leica DMIL LED Inverted Microscope and Leica DFC 295 Digital Microscope Color Camera (Leica Microsystems, Wetzlar, Germany).

### 2.5. Presto Blue Viability Assay

Human MSCs were plated at 90% confluence in 96-well plates, each plate containing unexposed and VP16 exposed cells, with 5 wells per group. Cells were exposed to 25 *μ*M VP16 or medium only control for 24 hours. After incubation, cells were washed 3 times and allowed for 0, 6, or 48 hours to recover. At each time point, viability was assessed using Presto Blue Cell Viability Reagent (Life Technologies, Carlsbad, California, United States) as per manufacturer's recommendation. Well ODs were blanked to wells containing medium and Presto Blue Cell Viability Reagent alone prior to analysis.

### 2.6. Etoposide Exposure

VP16 (Bristol-Myers Squibb, New York, New York, United States) was stored in 33.98 mM aliquots at −20°C and diluted to 25 *μ*M immediately prior to use in MSC medium (see Section 2.1).

### 2.7. Fluorescent Microscopy

Cells were plated at 90% confluence on glass coverslips in 24-well plates and then exposed to 25 *μ*M VP16 or medium alone for 24 hours. Following incubation, cells were washed three times and then allowed for 0, 6, or 48 hours to recover. At each recovery time, coverslips were washed with 1x PBS and then fixed for 6 hours in 4% paraformaldehyde. Following fixing, cells were permeabilized with 0.5% Triton X-100 and then treated with Image-iT FX Signal Enhancer (Life Technologies, Carlsbad, California, United States) for 20 minutes. Following a 20-minute block with 5% BSA, cells were incubated with *γ*-H2AX antibody (1 : 400 Dilution, Millipore, Billerica, Massachusetts, United States) overnight at 4°C. Following incubation, the cells were washed in 1x PBS and then incubated for 1 hour in Goat anti-Rabbit-FITC secondary antibody (1 : 200, Cell Signaling, Danvers, Massachusetts, United States) prior to mounting on coverslips with ProLong Gold Antifade Reagent with DAPI (Life Technologies, Carlsbad, California, United States). Antibodies were diluted in 5% BSA. Following staining, cells were imaged on a Zeiss LSM 510 Laser Scanning Confocal Microscope (Zeiss, Jena, Germany). *γ*-H2AX was quantitated as cells expressing 10 or more foci or manually counted at the 6-hour recovery time point to determine *γ*-H2AX on a per-cell basis. The 6-hour recovery time point was utilized for counting because foci were too abundant at 0 hours of recovery to discern individual foci.

### 2.8. RNA Isolation and qPCR

RNA was isolated using Qiagen RNeasy Minikit (Qiagen, Valencia, California, United States) and stored at −80°C prior to qPCR. RNA stock solutions were diluted to 50 ng/11 *μ*L concentration prior to qPCR analysis. One-step qPCR reactions were performed using QuantiTect SYBR Green RT-PCR Kit (Qiagen, Valencia, California, United States) and a 7500 Applied Biosystems Thermalcycler (Applied Biosystems, Foster City, California, United States). Primers sequences are indicated in Supplementary Table  1 in Supplementary Material available online at http://dx.doi.org/10.1155/2016/683861. PCR data were analyzed using the ΔΔCt method [20].

### 2.9. Cell Cycle Analysis

Following trypsinization, cells were fixed in 70% ethanol for 24 hours prior to cell cycle analysis. Once fixed, cells were washed in 1x PBS and then stained with propidium iodide staining solution (0.1% Triton X-100, 0.2 mg/mL RNaseA, and 0.02 mg/mL propidium iodide) for 30 minutes at room temperature. Cells were then resuspended in 1x PBS and ran on Beckman FACS Calibur (BD Biosciences, Franklin Lakes, New Jersey, United States). Analysis of cell cycle data was performed using FCS Express 4 Software (De Novo Software, Glendale, California, United States).

### 2.10. Western Blot Analysis

Protein was isolated from whole cell lysates prior to Western analysis using reducing conditions. Blots were probed using Rad51 (Cell Signaling, Danvers, Massachusetts, United States) and XRCC3 (Novus Biologicals, Littleton, Colorado, United States) antibodies and anti-rabbit-HRP (Cell Signaling, Danvers, Massachusetts, United States). Immobilon Western ECL reagents (EMD Millipore, Billerica, Massachusetts, United States) were used to develop membranes.

### 2.11. NHEJ and HR Reporter Assays

Functional contributions of NHEJ and HR to DSB repair were evaluated using reporter assays developed and kindly provided by Dr. Vera Gorbunova (University of Rochester, Rochester, NY). NHEJ and HR constructs are designed with a Pem1 adenoviral intron interrupting the reading frame of GFP. The Pem1 intron is flanked by I-SceI restriction sites that, when repaired by NHEJ or HR, restores the reading frame of GFP, resulting in expression which can be quantitated by flow cytometry along with a DsRed loading control to evaluate transfection efficiency. The total %GFP^+^/%DsRed^+^ gives the repair efficiency, a number that quantitatively reflects the degree to which MSCs have utilized NHEJ or HR to repair the reporter plasmid [21]. Cells were exposed to 25 *μ*M VP16 for 24 hours prior to isolation of cells for analysis as previously described [22]. Cells were nucleofected using an Amaxa Nucleofector (Lonza, Basel, Switzerland), program U-23. Cells were nucleofected with 2 *μ*g HR or 0.5 *μ*g NHEJ constructs and 0.5 *μ*g DsRed loading control. Prior to nucleofection, NHEJ and HR constructs were linearized with I-SceI (New England Biolabs, Ipswich, Massachusetts, United States) as previously described [22]. 10 *μ*g of EGFP-N1 or DsRed-Express-DR (Clontech, Mountain View, California, United States) plasmids were included as positive controls. Following nucleofection, cells were cultured for 72 hours and then evaluated for GFP and DsRed expression using FACS Fortessa flow cytometer.

### 2.12. Statistical Analysis

For Presto Blue viability analysis, two-way Student's *t*-tests were performed comparing untreated to treated cells at each recovery time. *γ*-H2AX quantitation and NHEJ/HR reporter assay data were evaluated by One-Way ANOVA with Holm-Sidak Post Hoc test. *γ*-H2AX data in Supplemental Figure  2 were evaluated using the Kruskal-Wallis test. Statistical significance was defined as *p* value ≤ 0.05 using SigmaPlot Version 11 (Systat Software Inc., San Jose, California, United States). Experiments have been repeated at least twice using cell lines derived from different patients and three times for surface marker phenotyping, Presto Blue, cell cycle analysis, *γ*-H2AX microscopy, and NHEJ/HR qPCR experiments. Error bars in all figures indicate standard error.

## 3. Results

### 3.1. MSC Characteristics with Passage* Ex Vivo*

**Figure 1:**
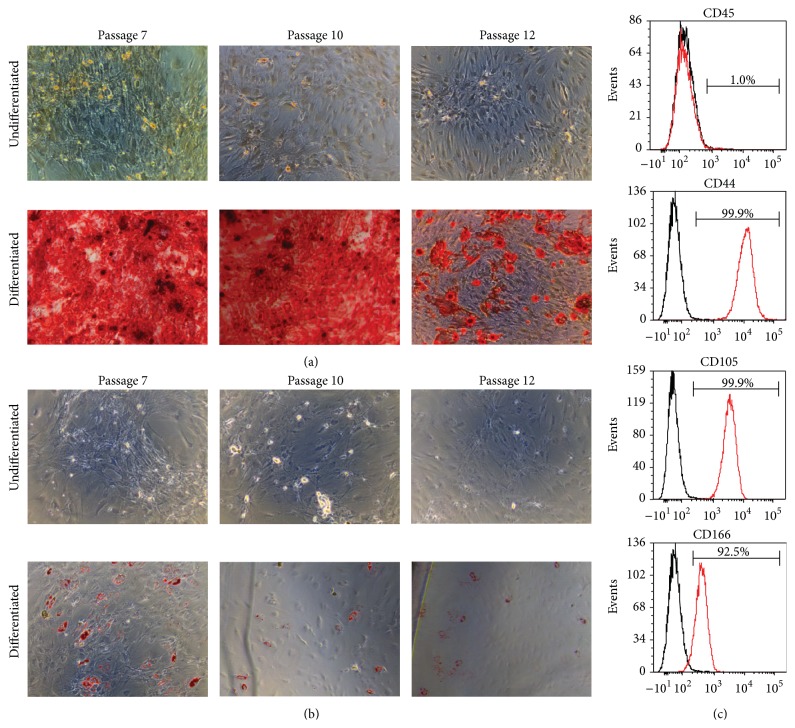
Differentiation and surface phenotype of bone marrow derived mesenchymal stem cells (MSCs). (a) Osteogenic and (b) adipogenic differentiation at various passages evaluated by alizarin red and oil red staining, respectively. (c) Flow cytometry detecting surface expression of MSC surface markers. Black lines represent isotype controls; red lines represent indicated surface markers.

Consistent with an MSC phenotype [23, 24], bone marrow derived MSCs showed an ability to perform both osteogenic and adipogenic differentiation (Figures 1(a) and 1(b)) and were CD45^−^CD44^+^CD105^+^CD166^+^ (Figure 1(c)). The adipogenic differentiation potential of MSCs appeared consistent up to passage 12 (Figure 1(b)). Previously, MSCs have been shown to display reduced osteogenic differentiation potential following prolonged passage* in vitro* [11, 12]. In addition, extensive culturing of MSCs has been shown to reduce the proportion of cells in S phase [25]. Consistent with these observations, we found that the osteogenic differentiation potential of passage 12 MSCs was reduced relative to less passaged cells (Figure 1(a)). The cell cycle distribution of MSCs was similar in passage 6 to passage 10 cells; however, the proportion of cells in S phase was reduced at passage 12 (Figure 3(a), untreated). These results indicate that our cells were characteristic of MSCs and displayed a functional phenotype consistent with extended passage* ex vivo* by passage 12.

In addition to differentiating into various mesenchymal lineages, MSCs functionally contribute to the regulation of the differentiation and proliferation of hematopoietic cells [26]. MSCs also provide chemotactic gradients that enable hematopoietic cell homing to the bone marrow [27], a process that we have previously shown to be negatively affected by chemotherapy exposure of bone marrow stromal cells and osteoblasts [28, 29]. A preliminary set of experiments did not provide evidence for passage related alterations of the ability of VP16 exposed MSCs to regulate the proliferation or chemotaxis of a stromal cell dependent murine pro-B cell clone or the differentiation of normal human CD34^+^ hematopoietic progenitor cells (data not shown).

### 3.2. Sublethal Concentrations of VP16 Induce Cell Cycle Arrest in MSCs Regardless of Passage

**Figure 2:**
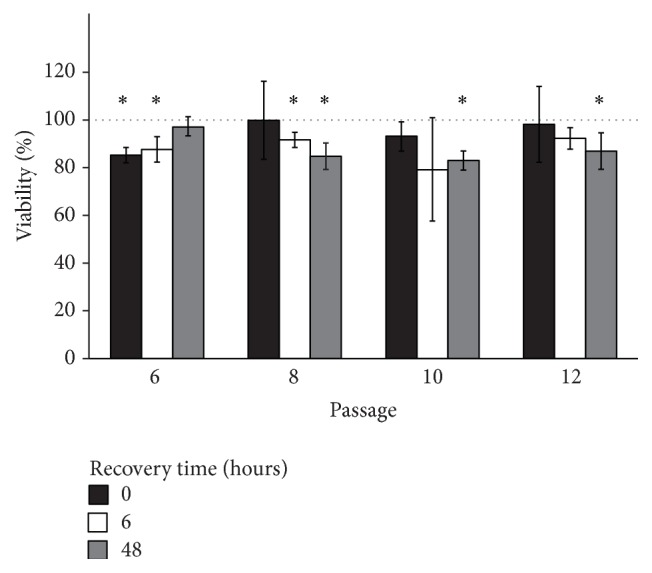
Viability of MSCs with passage following 24 hours of etoposide (VP16) exposure. MSCs at various passages were exposed to 25 *μ*M VP16 for 24 hours and then allowed for 0, 6, or 48 hours to recover in fresh medium before evaluation of viability by Presto Blue viability reagent. Values are reported relative to untreated controls. *∗* indicates a significant decrease in viability relative to untreated control, Student's *t*-test; *p*-value < 0.05.

To determine how MSCs respond to stress with successive passage* in vitro*, cells were exposed to VP16 for 24 hours at 90% confluence, then washed three times in complete medium, and allowed for 0, 6, or 48 hours to recover prior to analyses. Cells were exposed to VP16 at 90% confluence due to the cell cycle specific nature of both VP16 and HR mediated repair of DSBs. VP16 specifically induces DSBs during mitotic events [16]; therefore subconfluent cells were used in our model. 25 *μ*M VP16 was utilized because it was the highest concentration of VP16 that did not result in overt cell death up to 48 hours of recovery time (Figure 2 and data not shown) and therefore represented sublethal stress in our model. Exposure of MSCs to VP16 resulted in occasional statistically significant drops in viability; however no passage related trends were present. Consistent with the absence of passage related trends in the viability of MSCs following VP16 exposure, the abundances of proapoptotic (PUMA and NOXA) and antiapoptotic (BCL-XL and BCL-2) transcripts were similar with passage following VP16 stress (Supplementary Figure 1). These results suggest that MSCs do not display alterations in susceptibility to VP16 up to passage 12* in vitro*.

**Figure 3:**
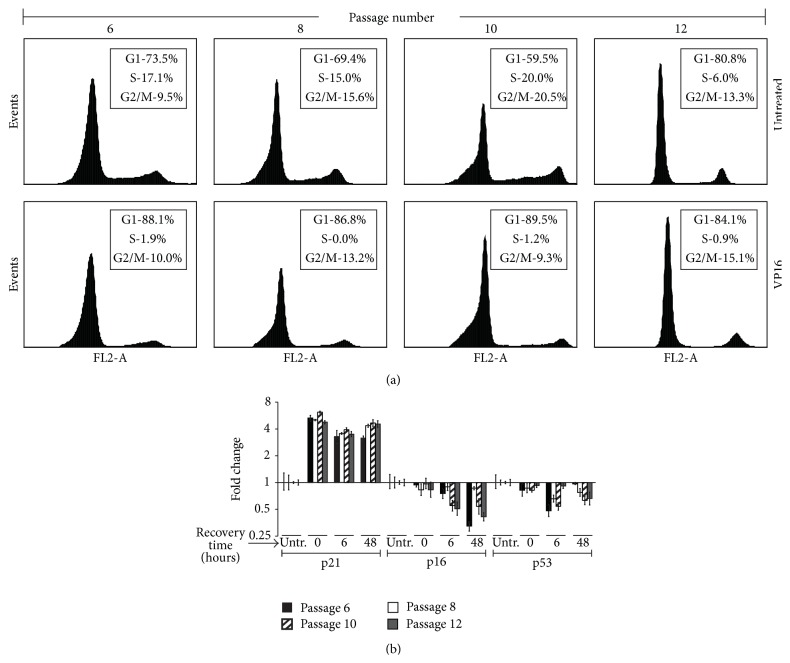
Effects of VP16 on cell cycle distribution and cell cycle inhibitor transcript abundance with passage. (a) Cell cycle distribution of MSCs at various passages exposed to 25 *μ*M VP16 or medium only control for 24 hours. (b) qPCR evaluation of cell cycle inhibitor mRNA expression following exposure of MSCs to 25 *μ*M VP16 for 24 hours followed by 0, 6, or 48 hours of recovery in fresh medium. Values are indicated as fold change relative to untreated control.

Exposure of MSCs to sublethal concentrations of VP16 resulted in a transcriptional induction of p21 which was similar among all passages (Figure 3(b)). p21 was elevated approximately 4- to 6-fold after 24 hours of exposure to VP16 and remained elevated following 48 hours of recovery. Consistent with increased p21, VP16 exposure resulted in a reduction in the proportion of cells in S/G2/M phase of cell cycle (Figure 3(a)). The transcriptional abundances of p16 and p53 were gradually decreased following exposure to VP16 (Figure 3(b)), suggesting that the observed G1 arrest was due to the earlier and relatively more robust induction of p21 following VP16 exposure. These results suggest that VP16 induces cell cycle arrest at all passages in* ex vivo* expanded MSCs.

### 3.3. *Ex Vivo* Passage of MSCs Does Not Alter Incidence or Repair Kinetics of DSBs following VP16 Exposure

**Figure 4:**
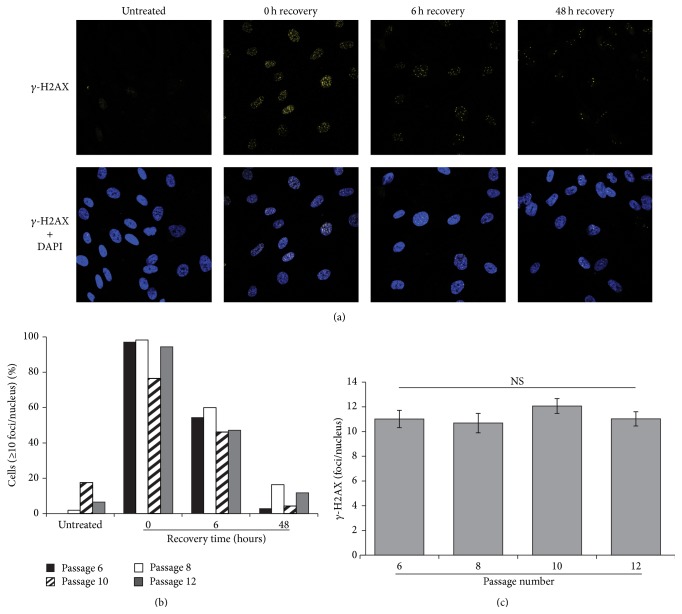
DNA double strand break incidence and repair kinetics in MSCs exposed to 25 *μ*M VP16. (a) Representative *γ*-H2AX staining (yellow) of Passage 6 MSCs exposed to VP16 for 24 hours followed by 0, 6, or 48 hours of recovery in fresh medium. *γ*-H2AX (Yellow) displays nuclear colocalization with DAPI (blue). (b) Percentage of MSCs displaying 10 or greater *γ*-H2AX foci at each time point at various passages. (c) Average number of *γ*-H2AX foci per cell at 6 hours of recovery time for passages 6 through 12. “NS” indicates no significant difference between any passage by One-Way ANOVA with Holm-Sidak Post Hoc test; *p*-value > 0.05.

To visualize DSBs in MSCs following VP16 exposure, immunofluorescent staining of *γ*-H2AX was utilized, as described previously [30]. MSCs displayed a large number of DSBs following 24 hours of exposure to VP16 (Figure 4(a)). After 48 hours of recovery time, the abundance of DSBs decreased, resulting in less than 20% of cells displaying 10 or more *γ*-H2AX foci (Figures 4(a) and 4(b)). The proportion of cells with 10 or more *γ*-H2AX foci did not appear influenced by passage number at any time point, suggesting that the repair kinetics of DSBs are similar among MSCs of all passages. To more accurately quantitate the number of DSBs on a per-cell basis, *γ*-H2AX foci were manually counted at 6 hours of recovery, showing no significant change with passage (Figure 4(c)). Based on these findings, it appears that the incidence and repair kinetics of DSBs following VP16 induced stress do not change with passage* in vitro*.

### 3.4. Alterations in NHEJ and HR Mediated Repair of DSBs with Passage and VP16 Exposure

**Figure 5:**
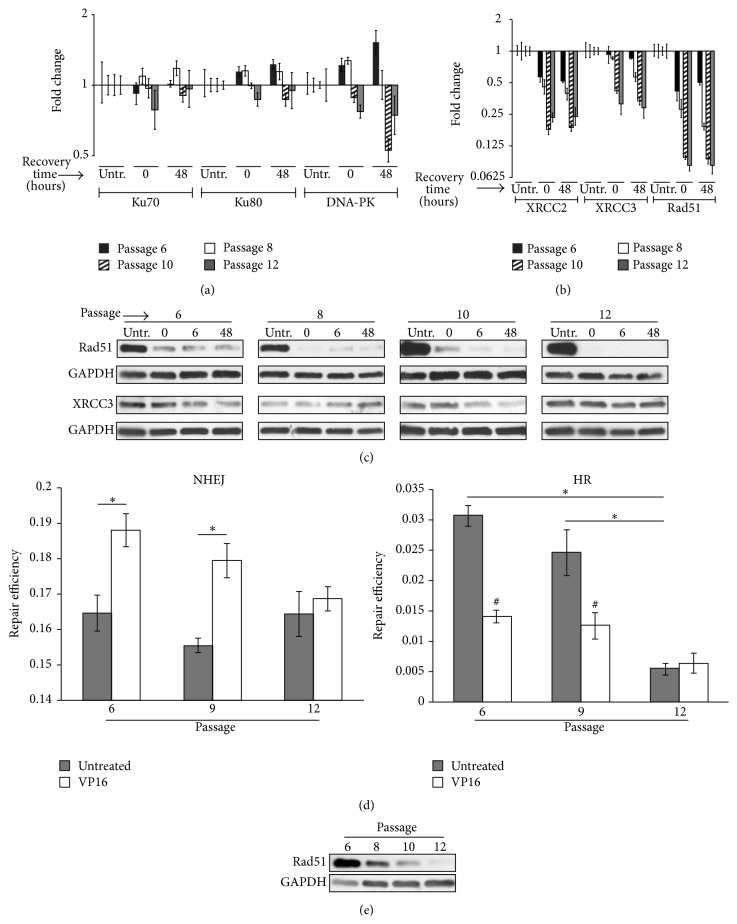
Nonhomologous end joining (NHEJ) and homologous recombination (HR) transcriptional, protein, and functional repair responses to 25 *μ*M VP16 with passage. qPCR evaluation of (a) NHEJ and (b) HR associated mRNA expression following exposure of MSCs to VP16 for 24 hours followed by 0 or 48 hours of recovery in fresh medium. Fold changes are indicated relative to untreated controls. (c) Western analysis of Rad51 and XRCC3 after VP16 exposure with passage. “Untr” indicates untreated cells. “0,” “6,” or “48” indicates recovery time (in hours) following 24 hours of exposure to 25uM VP16. (d) NHEJ and HR plasmid reporter assays for Passage 6, 9, and 12 MSCs exposed to VP16 or medium only control for 24 hours. (e) Western analysis of Rad51 in untreated MSCs with passage. # indicates significant change relative to untreated control; *∗* represents significant change relative to indicated group, One-Way ANOVA with Holm-Sidak Post Hoc test; *p*-value < 0.05.

To determine which repair pathways MSCs utilize to repair DSBs, as well as whether pathway dependence is altered by* in vitro* passage, transcriptional responses of NHEJ and HR associated genes were evaluated in our model. KU70, KU80, and DNA-PK were used to evaluate NHEJ, while XRCC2, XRCC3, and RAD51 were used to evaluate HR. Following VP16 stress, NHEJ associated transcripts remained relatively unaltered (less than 2-fold changes from baseline) and displayed no passage associated trends in abundance (Figure 5(a)). However, VP16 exposure reduced the abundance of HR associated transcripts, and this reduction was augmented with passage* in vitro* (Figure 5(b)). The decreased presence of HR transcripts following VP16 is consistent with the induction of cell cycle arrest displayed by our cells, given that HR can only be performed during S/G2 phases of the cell cycle [15]. The augmented decrease in HR associated transcripts with passage (following VP16 exposure) does not appear to be due to the reduced cycling-status of MSCs with extended passage, as the HR transcript decreases are present immediately following passage 6 while extended culture associated decreases in S/G2/M are not present until passage 12 (Figure 3(a), untreated). Consistent with qPCR results, protein abundance of Rad51 was decreased following VP16 stress at all passages. However, the abundance of XRCC3 was unaltered (Figure 5(c)). These data suggest that the reliance of MSCs on HR is reduced following VP16 stress and that there is altered double strand DNA repair pathway utilization with* in vitro* passaging of MSCs that precedes the changes in cell cycle distribution associated with* in vitro* culture.

To evaluate the functional contribution of NHEJ and HR to DSB repair in MSCs following VP16 exposure, NHEJ and HR plasmid reporter assays were utilized. NHEJ and HR reporter assays serve as a means to quantitatively evaluate the functional ability of cells to perform NHEJ or HR, reported as repair efficiency [22]. The average repair efficiency of NHEJ in untreated passage 6 cells was approximately 0.165, compared to 0.03 for HR, indicating that NHEJ is the predominant repair pathway of DSBs in MSCs, consistent with other cell types of the body [31] (Figure 5(d)). The repair efficiency of NHEJ in untreated cells remained consistent with passage of MSCs; however, the repair efficiency of HR was significantly reduced by passage 12 (Figure 5(d)). Coincident with reduced HR repair efficiency, untreated cells displayed reduced Rad51 protein abundance with passage (Figure 5(e)). Following VP16 exposure, passage 6 and 9 cells displayed significantly elevated repair efficiency for NHEJ, while the efficiency of HR was significantly reduced (Figure 5(d)). Interestingly, passage 12 cells did not display significant changes in repair efficiency following VP16 exposure relative to untreated controls (Figure 5(d)). These results indicate that NHEJ is primarily used to repair DSBs in MSCs and that NHEJ is increased in the context of VP16 stress while HR is decreased. However, once cells have undergone extended passage* in vitro*, MSCs are less able to utilize HR for repair, and DSB repair using NHEJ is not functionally increased following VP16 exposure. These data suggest that prolonged passage of MSCs* in vitro* can alter the ability to utilize NHEJ and HR following exposure to sublethal VP16 induced stress.

## 4. Discussion

The plasticity and immunomodulatory potential of MSCs have attracted attention regarding their application in cellular therapies for numerous diseases. The ability of MSCs to expand* in vitro* has further increased enthusiasm for clinical application, circumventing potential problems with the acquisition of sufficient cell numbers for transplantation. In addition, MSCs can be acquired from various tissues of the body [4], making it possible to utilize cells from a patient autologously, nullifying the risks of graft versus host disease. The ability of MSCs to expand* ex vivo* is beneficial towards their use clinically, highlighting the necessity of understanding how cells are changed during culture* in vitro*.

*In vitro* culture has been shown to epigenetically regulate gene expression in MSCs. Passage related increases in HDAC activity correlate with increased expression of HDAC4, HDAC5, and HDAC6, resulting in reduced H3 and H4 acetylation and reduced OCT4 expression [9]. Another study documented passage associated alterations in methylation at specific CpG sites, many of which regulate differentiation associated genes including RUNX3 [10], which has been implicated in osteogenesis [32]. In addition to alterations in gene regulation,* in vitro* passaging of MSCs reduces osteogenic differentiation capacity [11, 12]. Consistent with these observations, we showed a reduced ability of passage 12 MSCs to differentiate under osteogenic conditions relative to lower passages (Figure 1(a)), indicating that our cells had been cultured sufficiently to elicit changes in MSC function. However, we did not observe reduced adipogenic differentiation potential up to passage 12 (Figure 1(b)) which defined the endpoint of our model. These studies display important cellular characteristics that are altered with* ex vivo* passage, but little has been done to address how passaging alters their response to stresses resulting in DSB formation. Part of the reason DSBs are the most damaging of DNA lesions is the result of increased likelihood of erroneous repair, especially following NHEJ [33]. Erroneous repair is associated with increased cellular transformation, a phenomenon that has been documented with* in vitro* passage of MSCs [7, 8], alluding to the importance of maintaining not only survival, but genomic integrity of* ex vivo* expanded MSCs. The specific induction of DSBs by VP16 enabled us to specifically evaluate MSC responses regarding DSB repair. Although any type of DNA lesion can be harmful to a cell, DSBs are considered the most serious and potentially mutagenic [13], hence their focus in our investigation.

To model cellular stress as a consequence of DSB presence, we utilized sublethal concentrations of VP16 that displayed no passage associated changes in the susceptibility of MSCs to VP16 induced cell death (Figure 2). VP16 did not result in overt death but did increase p21 mRNA and elicited a reduction in the proportion of cells in S/G2/M phases of cell cycle indicating cell cycle arrest at all passages (Figures 3(a) and 3(b)). Although capable of initiating G1 arrest following cellular stress [34], we found p16 and p53 transcripts to be reduced by 6 hours of recovery time (Figure 3(b)). The earlier and more robust presence of p21 may have chiefly contributed to the induction of cell cycle arrest, consistent with previous observations in stressed MSCs [35]. These observations show that MSCs display signs of cellular stress in our model of VP16 exposure in a manner that is sublethal. The absence of cellular death in our model enabled us to evaluate the incidence, repair kinetics, and cellular repair pathways of DSBs within MSCs with passage.

Of the numerous types of DNA lesions, VP16 specifically generates DSBs [36]. Within minutes following the formation of a DSB within the nucleus, various kinases (including DNA-PK, ATM, and ATR) detect the presence of the break and phosphorylate histone 2A moieties. Phosphorylated histone 2A (*γ*-H2AX) present as nuclear puncta corresponding to an individual DSB that can be visualized by immunofluorescence [30]. When evaluating *γ*-H2AX foci at each time point after VP16 exposure, changes were not observed between passages (Figure 4(b)) suggesting that the rate by which MSCs repair VP16 induced DNA damage is not affected by passage* in vitro*. Notably, less than 20% of the cells contained 10 or more *γ*-H2AX foci by 48 hours of recovery (Figure 4(b)). The greatly reduced presence of DSBs following 48 hours of recovery time suggests that MSCs are capable of recovering from 25 *μ*M VP16 exposure, consistent with the sublethal nature of the model. These results indicate that the incidence and repair kinetics of DSBs following VP16 exposure were similar across passage number* in vitro*.

The resolution of DSBs following 48 hours of recovery from VP16 exposure suggests the intact presence of double strand DNA repair pathways within MSCs for all passages observed. DSBs are repaired through two major pathways: NHEJ and HR [15]. To elucidate the contribution of these pathways to repair with passage in the context of VP16 stress, transcriptional responses of a panel of NHEJ and HR genes were evaluated. Although NHEJ transcripts displayed little change, HR associated transcripts were reduced with VP16 exposure (Figures 5(a) and 5(b)). Evaluations of Rad51 and XRCC3 protein showed that although XRCC3 was unaltered by passage or VP16 exposure, Rad51 was reduced by VP16, consistent with qPCR results (Figure 5(c)). Although these results served as evidence for VP16 exposure and passage playing a role in the means by which MSCs repair DNA, qPCR cannot describe which pathways are being utilized functionally by MSCs and to what extent. To functionally and quantitatively evaluate the contribution of NHEJ and HR to DSB repair in VP16 stressed MSCs, plasmid based reporter assays were utilized. We showed that, at baseline, NHEJ was the primary DSB repair pathway utilized by MSCs (Figure 5(d)), consistent with most cell types of the body [17]. In lower passage cells (passages 6 and 9), NHEJ is increased while HR is decreased following VP16 stress (Figure 5(d)). However, in passage 12 cells, there was reduced presence of HR at baseline, possibly due to the reduced abundance of Rad51 (Figure 5(e)), and VP16 induced increases or decreases in NHEJ or HR (resp.) were not present (Figure 5(d)). These results suggested that, in lower passage cells, NHEJ is increased to repair DNA damage while HR is decreased; however, later passage cells are unable to increase NHEJ mediated repair following VP16 stress. Passaging and irradiation of fibroblasts have been shown to alter the abundance and localization of Ku70/80 [37], possibly playing a role in the inability of late passage MSCs to increase NHEJ efficiency following VP16 stress (Figure 5(d)). Quantitation of *γ*-H2AX foci in untreated cells with passage did not show significant changes (Supplementary Figure 2), suggesting that the genomic integrity of untreated cells is not affected by defects in HR repair with* ex vivo* expansion. In addition, the inability of passage 12 MSCs to increase NHEJ following VP16 exposure did not result in alterations of DSB presence or viability when compared to lesser passaged MSCs (Figures 2 and 4). These results are most likely due to the sublethal nature of our model. It is possible that, at higher concentrations of VP16, the consequences of less efficient DNA repair would affect the presence or repair kinetics of DSBs. However, due to the nature of our assays only evaluating living cells, the phenomenon would likely not be detected (a problem evaded by our use of nonlethal concentrations of VP16). Nevertheless, our results suggest a defective ability of MSCs to increase NHEJ, their primary repair pathway of DSBs, in the context of VP16 stress following successive passage* in vitro*.

Our results showing reduction in the efficiency of HR following* in vitro* culture of MSCs are consistent with previous findings in fibroblasts [38]. However, when Seluanov et al. evaluated NHEJ efficiency in fibroblasts with successive passaging (up to 70 population doublings), NHEJ efficiency was found to be decreased [39]. Our results with normal primary human MSCs did not show significant changes in NHEJ efficiency in untreated cells regardless of passage, possibly due to the fact that our cells were passaged to a lesser extent (passage 12). The results presented in this report contribute unique information by evaluating passage related changes in DSB repair in the context of cellular stress, a common circumstance that will be encountered when the ability to repair DNA is crucial. In addition, DSB repair being quantitatively evaluated in MSCs adds to our understanding of the characteristics of these clinically valuable stem cells. Given recent observations that NHEJ utilization varies by tissue type* in vivo* [31], the specific evaluation of DNA repair in human primary marrow derived MSCs is relevant to developing optimal models to expand cells* ex vivo* for diverse cellular therapies.

## 5. Conclusions

In conclusion, we have determined that extended culture of human primary bone marrow derived MSCs results in an inability to functionally increase NHEJ when encountering sublethal VP16 stress and reduced utilization of HR in the absence of stress. Given the necessity of* ex vivo* expansion of MSCs for use in cellular therapies, these results serve as a guideline for improving strategies to sufficiently expand MSCs without inducing culture associated alterations that could have negative effects on the ability of the cells to persist following transplantation. Both the inability of MSCs to increase NHEJ following VP16 stress and reduced osteogenic differentiation capacity were detected at passage 12 (Figures 5(d) and 1(a), resp.). Although we do not propose that these events are directly related, they highlight the possibility of utilizing a biomarker to determine when* ex vivo* expanded MSCs display impaired DSB repair abilities in the context of stress. The discovery of such biomarkers would enable screening for DNA repair deficiencies without the time, cell, and labor intensive requirements of performing plasmid based DSB repair assays prior to utilization of* ex vivo* expanded MSCs for cellular therapy.

## Supplementary Material

Supplementary materials for this manuscript include two figures and one table. Supplementary Figure 1 contains qPCR data evaluating the transcriptional abundance of pro-apoptotic (A) and anti-apoptotic (B) genes after VP16 exposure with passage. Supplementary Figure 2 shows the average number of Ƴ-H2AX foci (per nucleus) in untreated cells with passage, and Supplementary Table 1 contains all qPCR primer sequences used in this manuscript.
