# Supplementary material for: In Vitro Expansion of Bone Marrow Derived Mesenchymal Stem Cells Alters DNA Double Strand Break Repair of Etoposide Induced DNA Damage
Source: Stem Cells Int. 2016 Jan 6;2016:8270464. doi: 10.1155/2016/8270464 (PMC4736568; doi:10.1155/2016/8270464)
Supplement: Supplementary file 1 — Supplementary materials for this manuscript include two figures and one table. Supplementary Figure 1 contains qPCR data evaluating the transcriptional abundance of pro-apoptotic (A) and anti-apoptotic (B) genes after VP16 exposure with passage. Supplementary Figure 2 shows the average number of Ƴ-H2AX foci (per nucleus) in untreated cells with passage, and Supplementary Table 1 contains all qPCR primer sequences used in this manuscript. [file 8270464.f1.pdf]

Supporting Information

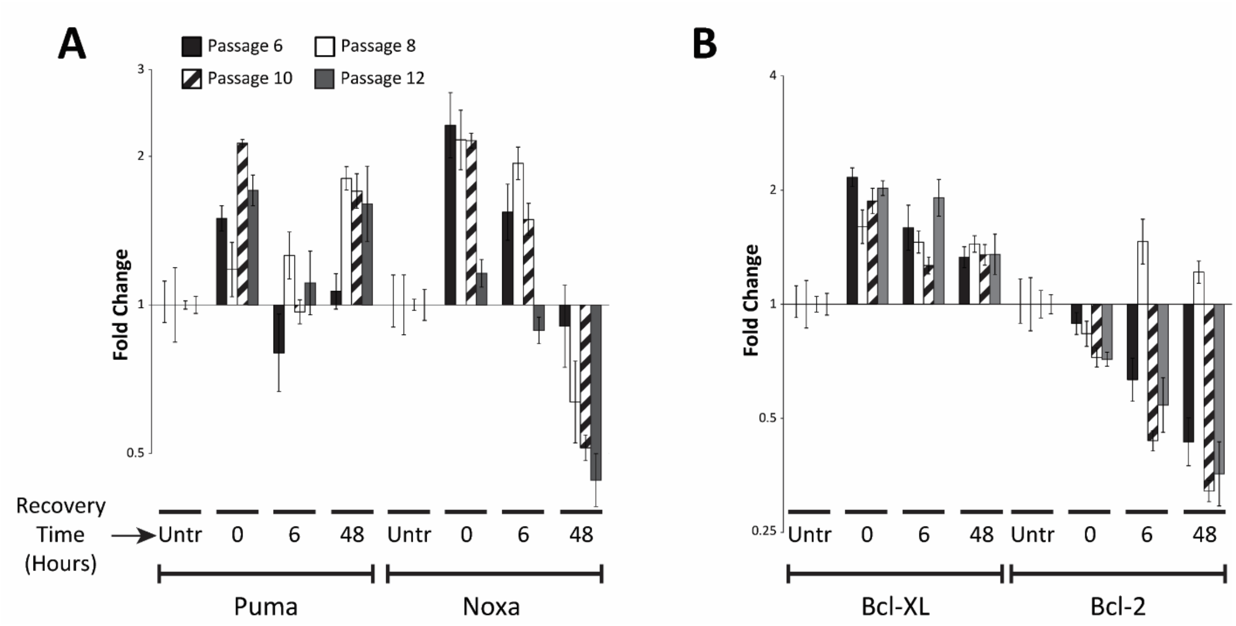

Supplementary Figure 1. Apoptosis associated transcriptional responses of MSCs following VP16 exposure with passage. (A) Pro-apoptotic and (B) anti-apoptotic mRNA expression evaluated by qPCR in MSCs exposed to 25μM VP16 for 24 hours followed by 0, 6, or 48 hours recovery in fresh medium. Fold changes are indicated relative to untreated controls.

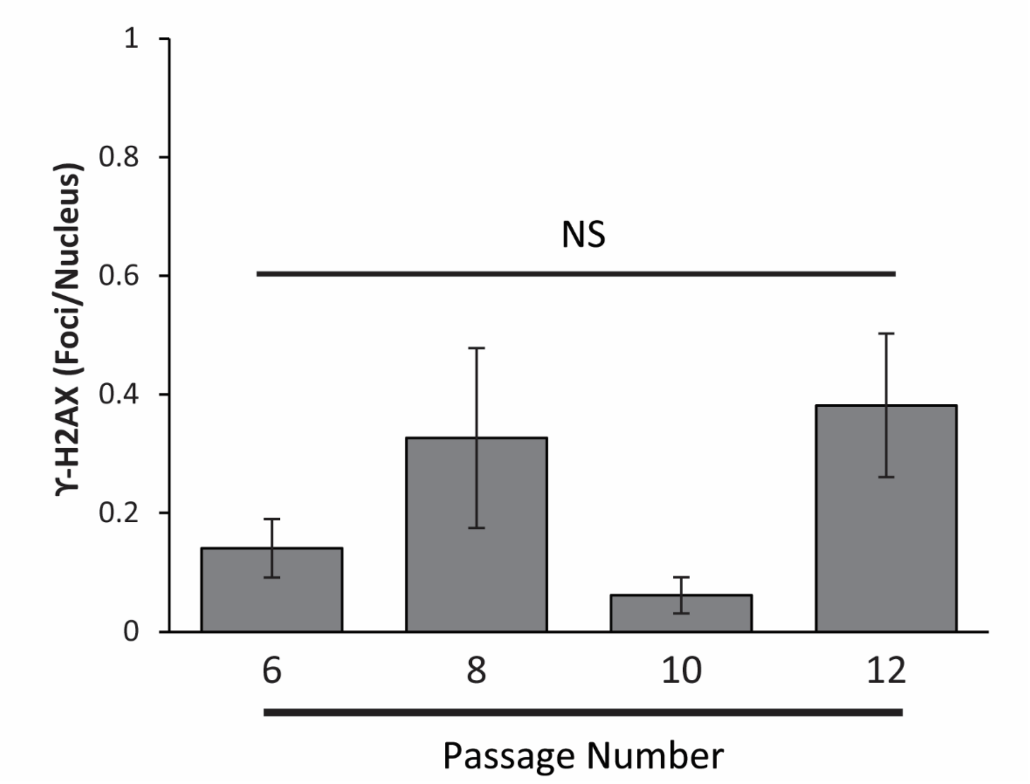

Supplementary Figure 2. Average number of ϒ-H2AX foci per cell in untreated MSCs for passages 6 through 12. “NS” indicates no significant difference between any passage by Kruskal-Wallis test, p-value > 0.05.

Supplementary Table 1. qPCR Primer sequences (5’ to 3’).

Gene	Forward	Reverse
GUSB	AAACGATTGCAGGGTTTCAC	CTCTCGTCGGTGACTGTTCA
CDKN1A (p21)	CCTGGCACCTCACCTGCTC	CGGCGTTTGGAGTGGTAGA
CDKN2A (p16)	GGGGTCGGGTAGAGGAGGT	CCGTGGAGCAGCAGCAGC
TP53 (p53)	GCACATGACGGAGGTTGTGAG	ATGGTGGTACAGTCAGAGCCAAC
KU70	CCAATAAAGCTCTATCGGGAAAC	TTTCTCCAGTATAATCTGACGACTCC
KU80	AAAATTAAAGACTGAGCAAGGGG	TAGAACACGGAAGTTTTCAGCAG
PRKDC (DNA-PK)	GCGAAGCACTGGCTTAGC	CTAACACTTCATCTTTAGGGACCC
XRCC2	GCAGTTGGTGAATGGCGTT	GCACAGGTGAATCTTCATCAGC
XRCC3	CGGCATCACTGAGCTGGC	AGCTCTCCTGGAACGTCAGTG
RAD51	GTCTCTCTGGCAGTGATGTCCT	TCTGTAAAGGGCGGTGGC
BBC3 (Puma)	AGACAAGAGGAGCAGCAGCG	CTGGGTAAGGGCAGGAGTCC
PMAIP1 (Noxa)	AGCTGGAAGTCGAGTGTGCTACT	GCAAGTTTTTGATGCAGTCAGG
BCL2 (Bcl-2)	TGTCGCAGAGGGGCTACG	GGATGCGGCTGGATGGG
BCL2L1 (Bcl-XL)	TCCAGGAGAACGGCGGC	GAGCCCAGCAGAACCACG
